# A conserved Chlamydiota-specific Type III Secretion System effector linked to stress response

**DOI:** 10.1099/mic.0.001545

**Published:** 2025-04-28

**Authors:** Thomas Kozusnik, Carole Kebbi-Beghdadi, Silvia Ardissone, Simone E. Adams, Gilbert Greub

**Affiliations:** 1 Institute of Microbiology, Lausanne University Hospital, Lausanne, Switzerland

**Keywords:** aberrant bodies, *Chlamydia trachomatis*, *Chlamydiota*, cystine knot, heat shock, HrcA regulation, iron starvation, persistence, protein misfolding, stress response, T3SS effector, *Waddlia chondrophila*

## Abstract

Despite broad genetic variability, members of the *Chlamydiota* phylum share a crucial stress response phenotype, the formation of aberrant bodies. However, how this response operates upon exposure to different kinds of stressors is still largely unknown. In *Waddlia chondrophila*, *wcw_0502* RNA levels are upregulated in aberrant bodies induced by iron starvation. Wcw_0502 is a putative type III secretion system (T3SS) effector and has a homologue in every known chlamydial species, regardless of their host. However, the upregulation of the *wcw_0502* gene expression upon iron starvation is not conserved in other chlamydial species such as *Chlamydia trachomatis*, *Chlamydia pneumoniae*, *Simkania negevensis* or *Estrella lausannensis*. Moreover, among all the stressors examined, only heat shock induced a strong upregulation of *wcw_0502* and its *C. trachomatis* homologue, *ctl0271*. A Controlling Inverted Repeat of Chaperone Expression sequence is present in the promoter region of *wcw_0502* and its homologues. We hypothesized that in the absence of stress, the conserved repressor HrcA, in association with the Hsp60 chaperone, binds this sequence and represses transcription. A decreased occupancy of HrcA and Hsp60 at the *wcw_0502* promoter region was observed in aberrant bodies induced by iron starvation when compared to reticulate bodies, which may lead to *wcw_0502* upregulation. The precise function of this newly described T3SS effector is still unclear. A cystine knot-like domain, a structural feature never described before in bacterial proteins, was found in the C-terminal region of Wcw_0502. This structure is described as highly resistant to proteolytic, chemical and thermic stressors, an advantageous property for a secreted protein with an increased production during stresses that impact protein integrity.

## Introduction

The *Chlamydiota* phylum consists of obligate intracellular bacteria known for their biphasic developmental life cycle and their broad host range. This phylum includes the *Chlamydiaceae* family with notorious agents of human diseases such as *Chlamydia trachomatis* and *Chlamydia psittaci* [1,2]. During the last decades, several new species were added to the phylum [3]. These species belong to new families and are referred to as *Chlamydia*-related bacteria (CRB). Unlike *Chlamydiaceae*, whose main hosts are vertebrates, CRB have a much broader host range and a larger genome [4]. For example, the genome of *C. trachomatis* is ~1.03 Mbp, while *Waddlia chondrophila*, a CRB from the *Waddliaceae* family associated with spontaneous abortion in humans and ruminants, has a genome size of 2.11 Mbp [5,8].

Despite differences in their metabolic capacities or their host range, all members of the *Chlamydiota* phylum share a common biphasic lifecycle [9,10]. The infectious form, elementary bodies (EBs), can penetrate the host cell using a mechanism resembling endocytosis. The endocytic vacuole, termed inclusion, surrounds the invading bacteria and protects them from being killed. The transition from EBs to the replicative form, reticulate bodies (RBs), will then occur. The inclusion also provides the bacterium with an appropriate environment for proliferation, leading to the exponential multiplication of RBs [9,11]. Following the replication phase, RBs transition back to EBs, and the infection cycle ends with the release of infectious progeny by either host cell lysis or exocytosis [9,10].

While *C. trachomatis* and *W. chondrophila* share a biphasic lifecycle and rely on a type III secretion system (T3SS) to hijack host cell processes, they exhibit notable differences in their interactions with host organelles. *C. trachomatis* inclusions predominantly interact with the endoplasmic reticulum and Golgi apparatus, acquiring lipids and nutrients for replication. In contrast, *W. chondrophila* inclusions recruit host cell mitochondria, potentially exploiting them for energy metabolism by using lipids and ATP from the host cell [12,13]. Another characteristic shared by the members of the *Chlamydiota* phylum is the phenotype observed during periods of stress, where a halt of division occurs and RBs grow in size, becoming less metabolically active [14,15]. The development of these aberrant bodies (ABs) allows survival during stressful conditions, such as a lack of nutrients or an antibiotic treatment. ABs can revert to RBs and resume the lifecycle once the stressor is no longer present.

ABs have been described in members of multiple chlamydial families and upon exposure to various types of stress, including iron starvation, peptidoglycan (PG) synthesis interference, heat shock, protein synthesis inhibition, exposure to glycopeptide antibiotics or response to interferon-gamma [13,18]. It was recently shown in *W. chondrophila* that different types of stress lead to various phenotypes of ABs [16]. These phenotypic differences include AB size, AB number per inclusion and inclusion size. ABs were also observed *in vitro* in human pneumocytes infected with *E. lausannensis* [19]. In addition they were also observed by electron microscopy during *in vivo* infection with *Chlamydia pecorum* and *Chlamydia suis* in mice and pigs [20,22]. This mechanism of persistence, through the static survival of bacteria and their reactivation upon stress clearance, is believed to be linked to recurrent and chronic infections in patients. However, despite the conservation of this phenotype across the phylum, the mechanisms underlying AB formation are still unclear.

Two previously reported global transcriptomic studies compared the gene expression between RBs and ABs induced by iron starvation during *in vitro* infection with either *C. trachomatis* or *W. chondrophila* [23,24]. Although these studies were performed in similar conditions, there were no obvious similarities in the differentially expressed genes between the two species.

In a previous study, we identified *W. chondrophila* genes upregulated in iron-starved ABs when compared to RBs [23], a clinically relevant condition, as iron starvation is a key host immune response to infection [25,27]. Building on this, we aimed to identify a potential genomic trigger of AB formation during iron starvation that is conserved across the different members of the *Chlamydiota* phylum. We focused on *W. chondrophila* genes upregulated by iron starvation that also have a homologue in *C. trachomatis*. Among them, *wcw_0502* was selected due to its strong conservation and its genomic organization with *wcw_0501* and *wcw_0503*, which is highly preserved across the phylum. By analysing the expression levels of *wcw_0502* in ABs induced by various stressors, as well as *wcw_0502* homologues in multiple chlamydial species, this study highlights the complexity of a seemingly conserved stress response mechanism. These findings underline the importance of combining molecular biology, cell biology and genomics as well as applying these approaches to various members of the *Chlamydiota* phylum to understand how aberrant bodies contribute to pathogen survival during persistence.

## Methods

### Antibodies, reagents and drugs

Polyclonal antibodies against Wcw_0502 were obtained by the immunization of rabbits with the purified protein (see below) (Eurogentec, Belgium). Mouse anti-V5 antibodies were obtained from Thermo Fisher Scientific (USA). Goat anti-*C. trachomatis* major outer membrane protein (MOMP) was obtained from Lifespan Bioscience (LS-C55983, USA). Mouse anti-*W. chondrophila* antibodies were homemade. Goat anti-rabbit HRP was from Promega (USA). Secondary anti-mouse, anti-donkey and anti-rabbit antibodies conjugated to Alexa-488 or Alexa-594 as well as the Texas red-conjugated concanavalin A were purchased from AppliChem (Germany). Clavulanic acid, deferoxamine, mecillinam, novobiocin, penicillin, phosphomycin, piperacillin, teicoplanin and 2,2′-bipyridyl were purchased from Sigma-Aldrich (USA). Human recombinant IFNγ was purchased from Gibco (USA). Vancomycin was obtained from AppliChem. MP265 was purchased from American Custom Chemicals Corporation (San Diego, CA, USA). All drugs were diluted in deionized water, except MP265, which was diluted in DMSO (AppliChem) and 2,2′-bipyridyl in ethanol.

### Bacteria and host cell culture

*W. chondrophila* strain ATCC VR-1471 was maintained in *Acanthamoeba castellani* strain ATCC 30010 at 32 °C in 25 cm^2^ flasks containing 6 ml of peptone–yeast–extract–glucose broth. After 7 days of co-culture, bacteria were isolated by filtering the suspension through a 5-µm filter. The filtrate was then diluted at the appropriate dilution in Dulbecco’s Modified Minimal Essential Medium (DMEM) (GE Healthcare, USA) to infect McCoy cells (strain ATCC CRL-1696) or HeLa cells (strain ATCC CCL-2, for IFNγ treatment).

*C. trachomatis* LGV2 (strain ATCC 902B) was stored in Sucrose-Phosphate buffer with Glutamine (SPG: 220 mM sucrose, 17 mM Na2HPO4, 2.6 mM NaH2PO4, 19.5 mM l-glutamic acid, pH 7.4) at −80 °C.

McCoy and HeLa cells were cultivated at 37 °C with 5% CO_2_ in DMEM supplemented with 10% FBS (GE Healthcare, USA).

### Infection

McCoy or HeLa cells were grown in 25 cm^2^ flasks (Techno Plastic Products, Switzerland) or 24-well plates at 37 °C in the presence of 5% CO_2_. Confluent cell cultures were infected either with *C. trachomatis* (MOI 0.1–1) or *W. chondrophila* (MOI 0.1–1). Cells were centrifuged for 10 min at 1,790 ***g*** for *W. chondrophila* or 900 ***g*** for *C. trachomatis* at room temperature (RT), incubated at 37 °C in the presence of 5% CO_2_ for 15 min for *W. chondrophila* and 30 min for *C. trachomatis,* before the replacement of the culture medium to synchronize infections and further incubation at 37 °C. 0 h post-infection (hpi) corresponds to the time when the infectious medium is replaced. For DNA and RNA extraction as well as protein analysis, cells were scraped at appropriate time points post-infection. Adherent cells on glass coverslips were used for immunofluorescence experiments.

### Stress treatment on infected cells

Experiments with stressors were performed in McCoy cells. Treatment was applied at 8 hpi, and infected cells were harvested at 24 hpi. For the treatment with human recombinant IFNγ, HeLa cells were used. HeLa cells were pre-treated 24 h prior to infection and harvested at 48 hpi. The concentration of the drugs can be seen in Table 1. Heat shock was modified from Huang *et al*. [28]. Briefly, McCoy cells, infected with either *W. chondrophila* or *C. trachomatis*, were incubated at 37 °C for 16 h and then either left untreated at 37 °C or moved to 42 °C for 30 min or 4 h before harvesting.

**Table 1.** Drugs used to induce aberrant bodies in different chlamydial species

Drug	Mode of action	Concentration used
2,2′-bipyridyl (BPDL)	Binds Fe2+ and Fe3+	*W. chondrophila*: 75 µM*C. trachomatis*: 100 µM*C. pneumoniae*: 150 µM*E. lausannensis*: 100 µM*S. negevensis*: 150 µM
Clavulanic acid	*β*-lactamase inhibitor	*W. chondrophila*: 900 ng/mL*C. trachomatis*: 900 ng ml^−1^
Heat shock	Heat shock	*W. chondrophila*: 42 °C (30 min/4 h)*C. trachomatis*: 42 °C (30 min/4 h)
Interferon gamma (Recombinant human)	Immune responseTryptophan degradation	*W. chondrophila*: 200 ng/mL*C. trachomatis*: 200 ng ml^−1^
Mecillinam	Targets Pbp2	*W. chondrophila*: 200 ng/mL*C. trachomatis*: 200 ng ml^−1^
MP265	Targets MreB	*W. chondrophila*: 100 µM*C. trachomatis*: 75 µM
Novobiocin	Targets DNA gyrase subunit B	*W. chondrophila*: 450 µM
Penicillin G	Targets Pbp2/Pbp3/FtsI/AmiA	*W. chondrophila*: 1,000 µg ml^−1^
Phosphomycin	Targets MurA	*W. chondrophila*: 500 µg ml^−1^
Piperacillin	Targets Pbp3/FtsI	*W. chondrophila*: 500 µg ml^−1^
Teicoplanin	Targets d-Ala-d-Ala moiety of peptidoglycan	*W. chondrophila*: 250 µg ml^−1^
Vancomycin	Targets d-Ala-d-Ala moiety of peptidoglycan	*W. chondrophila*: 500 µg ml^−1^

**Table 2.** Genes potentially involved in iron starvation-induced aberrant bodies List of most upregulated *W. chondrophila* genes, which have a *C. trachomatis* homologue, identified by RNA sequencing comparing (I) aberrant bodies induced by iron starvation vs. (II) reticulate bodies.

*W. chondrophila* locus	Gene name	Protein function	BPDL vs. untreated fold change (RNAseq)
*wcw_1342*	groEs1	Heat shock co-chaperone	4.17
*wcw_0502*		Conserved hypothetical protein	3.50
*wcw_0669*	dnaB	Putative replicative DNA helicase	3.43
*wcw_0883*	secA	Hypothetical protein translocase	3.18
*wcw_1848*	groEs3	Heat shock co-chaperone	2.69
*wcw_0542*	fabG	3-Oxoacyl-acyl carrier-protein reductase	2.68
w*cw_1636*	hrcA	Heat-induced transcription repressor	2.57

### Gene expression

Infected McCoy or HeLa cells were grown in T25 flasks (Techo Plastic Products, Switzerland) and collected with their culture supernatants in TRIzol (AmbionR, Life Technologies, Thermo Fisher Scientific). RNA was extracted as described in Chomczynski and Mackey [29]. Random primers and the GoScript Reverse Transcription Kit (Promega, Switzerland) were used to produce cDNA, which was analysed by quantitative PCR (qPCR) using I Taq SYBRGreen technology (BioRad, Switzerland). qPCR was performed with 4 µl of cDNA diluted 1/5 and with primers whose concentrations are listed in Data S10, available in the online Supplementary Material. The thermocycler programme was as follows: 3 min at 95 °C, 45 cycles of 15 s at 95 °C and 1 min at 60 °C. Additionally, the melting curve was obtained (15 s at 95 °C, 1 min at 55 °C, with a+0.5 °C increment up to 65 °C and 15 s at 95 °C). ΔΔCt calculations were applied to calculate the fold change using the 16S rRNA gene as an endogenous control. In stress conditions, results were standardized to the untreated controls. For the lifecycle kinetics, each value was standardized with the 24 hpi timepoint as previously published [23]. Prism 9 for Windows (GraphPad software) was used to build the graphs.

### *Yersinia enterocolitica* type III secretion assay and immunoblot

T3SS-dependent secretion of Wcw_0502 and CTL0271 was assessed using *Y. enterocolitica* as a heterologous system, as described in Da Cunha *et al.* [30]. Full-length CTL0271 or Wcw_0502 with a C-terminal V5 tag were cloned into pLJM3 Data S11) under control of the *yop* promoter and transformed into two *Y. enterocolitica* strains, one with functional T3SS machinery (ΔHOPEMT) and one with dysfunctional T3SS machinery (ΔHOPEMT δYscU). Bacteria were grown, with shaking (200 r.p.m.), in BHI medium supplemented with 20 mM sodium oxalate, 0.4% glucose and 20 mM MgCl_2_ for 2 h at 27 °C and then moved to 37 °C in a shaking water bath. At 37 °C, the *yop* regulon is activated and secretion of T3SS effectors occurs. After 4 h at 37 °C, the OD_600_ was measured, and bacteria were pelleted (centrifugation for 1 min at 13,000 ***g***). Bacterial pellets were resuspended in SDS loading buffer (Thermo Fisher, USA). Proteins in the supernatant were precipitated overnight at 4 °C with trichloroacetic acid and then pelleted. Pellets were then washed with acetone and resuspended in Laemmli buffer with *β*-mercaptoethanol (BioRad). Both fractions were normalized according to their OD_600_ values and analysed by SDS-PAGE. Briefly, samples were incubated for 5 min at 95 °C while shaking, ran through SDS-PAGE on a 10% polyacrylamide Mini-PROTEAN precast gels (BioRad) and transferred onto a nitrocellulose membrane. Membranes were blocked by 2 h incubation with 5% non-fat dry milk in Tris-buffered saline with 0.05% Tween 20 (TBS), washed three times with TBS, 0.5% milk and incubated overnight at 4 °C with primary antibodies. After three subsequent washes with TBS, 0.5% milk, membranes were incubated 1 h at RT with goat anti-rabbit IgG-HRP (BioRad) diluted 1/3,000 in TBS, 0.5% milk. Immunoblots were processed with ECL™ Prime Western Blotting Detection Reagent (Amersham, GE Healthcare) and analysed on an ImageQuant LAS4000 mini (Amersham, GE Healthcare). Proteins of interest were detected using a monoclonal mouse anti-V5 antibody (Invitrogen, Thermo Fisher) (1/5,000 dilution). The presence of MreB in the supernatant was determined to evaluate unwanted bacterial lysis using a homemade rabbit polyclonal antibody (1/5,000 dilution).

### Cloning, expression and purification of Wcw_0502 for antibody production

The ORF of *wcw_0502* was amplified by PCR and cloned into the pET28 vector (Novagen EMD Chemicals Inc., Germany) (Data S11). This vector allows the protein of interest to be expressed in *Escherichia coli* BL21 with a C-terminal 6xHis tag. Protein expression was induced over 2.5 h with 1 mM IPTG (Qbiogen, Switzerland). Wcw_0502 was then purified under non-denaturing conditions as previously described [31] and concentrated using an Amicon Ultra 15 3K device (Millipore). Proteins were then dialysed using a Slide-A-Lyzer dialysis cassette (Thermo Fisher) to decrease the level of imidazole. Final protein concentrations were determined using the Bradford Quick Start™ assay (Biorad). Purified proteins were sent to Eurogentec (Seraing, Belgium) for rabbit immunization.

### Wcw_0502 protein quantification

*W. chondrophila-*infected McCoy cells and culture supernatants were harvested at different time points ranging from 0 to 144 hpi. 2,2-Bipyridyl (BPDL)-treated and untreated samples were collected at 24 hpi. For each sample, a 50 µl aliquot was reserved for DNA extraction with the Wizard SV Genomic DNA Extraction Kit (Promega) to assess genome copy number using a *W. chondrophila-*specific qPCR [32]. Cells and bacteria from one T25 flask were pelleted by centrifugation at 12,000 ***g*** for 5 min. The pellets were washed with PBS and resuspended in 500 µl of Laemmli buffer with *β*-mercaptoethanol (BioRad). Proteins were then analysed by SDS-PAGE and immunoblotting as described above with the homemade polyclonal rabbit anti-Wcw_0502 antibody diluted 1/3,000 as the primary antibody. Immunoblots were visualized with ECL™ Prime Western Blotting Detection Reagent (Amersham, GE Healthcare), and images were acquired on an ImageQuant LAS4000 mini (Amersham, GE Healthcare). Signal intensities were measured with ImageJ and then normalized to the genome copy number. For the iron starvation experiment, the result was standardized to the untreated control. For Wcw_0502 protein expression, the result was standardized to the 24 hpi timepoint as previously published [23].

### Assessment of disulphide bond presence within the cystine knot-like domain of Wcw_0502

TOP10 thermocompetent *E. coli* were transformed with a pBAD22-*wcw_0502* plasmid encoding either WT *wcw_0502* or with mutants containing all six cysteines from the putative cystine knot mutated into alanines or only three cysteines mutated to alanines (see Data S6 and S7 for details on cloning and sequences). These *E. coli* clones were grown at 37 °C in Luria–Bertani medium and supplemented with ampicillin, 100 µg ml^−1^ (Darmstadt, Germany) to an OD_600_ of about 0.8. 3 ml of each culture was centrifuged at 10,000 ***g*** for 1 min, supernatant was removed and bacterial pellets were resuspended in Laemmli buffer with *β*-mercaptoethanol (BioRad). For the DTT-treated samples, DTT 10 mM was added and incubated for 10 min at 37 °C right before running the SDS-PAGE and Western blot analysis as described above.

### Chromatin immunoprecipitation followed by qPCR (ChIP-qPCR)

Twenty-five cm^2^ flasks with confluent McCoy cells were infected or not with *W. chondrophila* for 24 hpi. Infected cells were either treated with 2,2-bipyridyl at 8 hpi or left untreated before transfer to 50 ml falcons. Crosslinking was performed with 1% formaldehyde and 10 µM of sodium phosphate (pH 7.6) for 10 min at RT followed by 30 min on ice [33]. Following crosslinking, samples were washed twice in PBS before re-suspension in TES buffer (10 mM Tris-HCl pH 7.5, 1 mM EDTA, 100 mM NaCl) containing 10 mM DTT (AppliChem) and incubated for 10 min at 37 °C. Samples were centrifuged and re-suspended in a Ready-Lyse Lysozyme Solution (LGC Biosearch, UK), according to the manufacturer’s instructions.

The lysates were then sonicated on ice with 10 cycles of 30 s to shear DNA and obtain fragments of length ranging from 300 to 700 bp. Samples were centrifuged at 14,000 ***×*** r.p.m. for 2 min, and the supernatants were collected and diluted to 1 ml in ChIP buffer (0.01% SDS, 1.1% Triton X-100, 1.2 mM EDTA, 16.7 mM Tris-HCl pH 8.1 and 167 mM NaCl) with protease inhibitors (Roche, Switzerland). The diluted supernatants were pre-cleared with 150 µl of Protein-A agarose (Roche) for polyclonal rabbit anti-HrcA or with protein-G agarose (Roche) for polyclonal mouse anti-Hsp60. The pre-cleared supernatants were then incubated overnight at 4 °C with 2 µl of specific antibodies.

The immuno-complexes were captured by incubation for 2 h at 4 °C with Protein-A/G agarose pre-saturated with BSA and then washed once with low salt washing buffer (0.1% SDS, 1% Triton X-100, 2 mM EDTA, 20 mM Tris-HCl pH 8.1 and 150 mM NaCl), once with high salt washing buffer (0.1% SDS, 1% Triton X-100, 2 mM EDTA, 20 mM Tris-HCl pH 8.1 and 500 mM NaCl), once with LiCl washing buffer (0.25 M LiCl, 1% NP-40, 1% deoxycholate, 1 mM EDTA and 10 mM Tris-HCl pH 8.1) and twice with TE buffer (10 mM Tris-HCl pH 8.1 and 1 mM EDTA). The complexes were then eluted, twice, with 250 µl of elution buffer (SDS 1%, 0.1 M NaHCO_3_, freshly prepared) and incubated overnight at 65 °C with 300 mM NaCl to reverse the crosslinks.

The samples were then treated with 2 µg of Proteinase K for 2 h at 45 °C in 40 mM EDTA and 40 mM Tris-HCl (pH 6.5). DNA was extracted using phenol/chloroform/isoamyl alcohol (25:24:1), ethanol-precipitated using 20 µg of glycogen as carrier and finally re-suspended in 100 µl of water.

Amplification of the Controlling Inverted Repeat of Chaperone Expression (CIRCE) sequences in the known promoter region of HrcA and in the intergenic region of *wcw_0502* and *wcw_0503* was performed by qPCR using the QuantStudio 3 Real-time PCR System (Applied Biosystems) and the primers described in Data S10. The input percentage method as described in Lin *et al.* was used to assess the enrichment of the CIRCE sequences in the IP samples vs. the mock controls by applying the following equations: ΔCt [normalized ChIP] = (Ct [ChIP] − (Ct [Input] − Log2 (Input Dilution Factor); Input % = 100/2 ^ΔCt [normalized ChIP]^ [34]. We then divided the enrichment value of the RB sample with one of the AB samples to obtain the fold change value.

### Immunofluorescence and confocal microscopy

Cells seeded on glass coverslips were infected in a 24-well plate (Corning, Sigma-Aldrich) as described above. At appropriate time, cells were fixed with ice-cold methanol for 5 min at –20 °C, washed three times with PBS and incubated for at least 2 h in blocking solution (PBS, 0.01% NaN_3_ and 1% BSA) at 4 °C. Primary antibodies were applied for 2 h at RT (1/2,000 dilution for all). After three washing steps in PBS with 0.1% saponin, coverslips were incubated with various combinations of the following antibodies depending on the experiment: 1/500 for Alexa-fluor conjugated secondary antibodies, 1/3,000 for DAPI dilactate (4′,6-diamidino-2-phenylindole dihydrochloride, Molecular Probes, Thermo Fisher Scientific) and/or 1/50 for Texas Red-conjugated concanavalin A. Dilution was performed in blocking solution. Secondary antibodies were applied for 1 h at RT. After two washes with PBS 0.1% saponin, one with PBS alone and one with deionized water, the coverslips were mounted onto glass slides using Mowiol (Sigma-Aldrich). Cells were observed under a confocal microscope (LSM 900, Zeiss, Germany).

## Results

### *wcw_0502* is highly upregulated in iron-starved ABs compared to RBs

The dynamic changes in gene regulation that occur in *W. chondrophila* ABs undergoing stress induced by iron starvation were previously reported by our group [23]. In that work, an RNA-sequencing approach was used to compare the gene expression of *W. chondrophila* grown in HEp-2 cells in three conditions: RBs (collected 24 hpi), EBs (collected 72 hpi) and ABs (induced with 75 µM 2,2′-bipyridyl eight hpi and collected 24 hpi). To identify candidate genes potentially involved in the formation of iron-starved ABs, we selected the most upregulated genes in ABs, which share a homologue in *C. trachomatis*. Seven genes (*wcw_1342*, *wcw_0502*, *wcw_0669*, *wcw_0883*, *wcw_1848*, *wcw_0542* and *wcw_1636*) with a significant fold change difference (ranging from 2.68- to 4.17-fold increase in ABs compared to untreated RBs) were selected for further analysis (see Table 2).

**Fig. 1.**
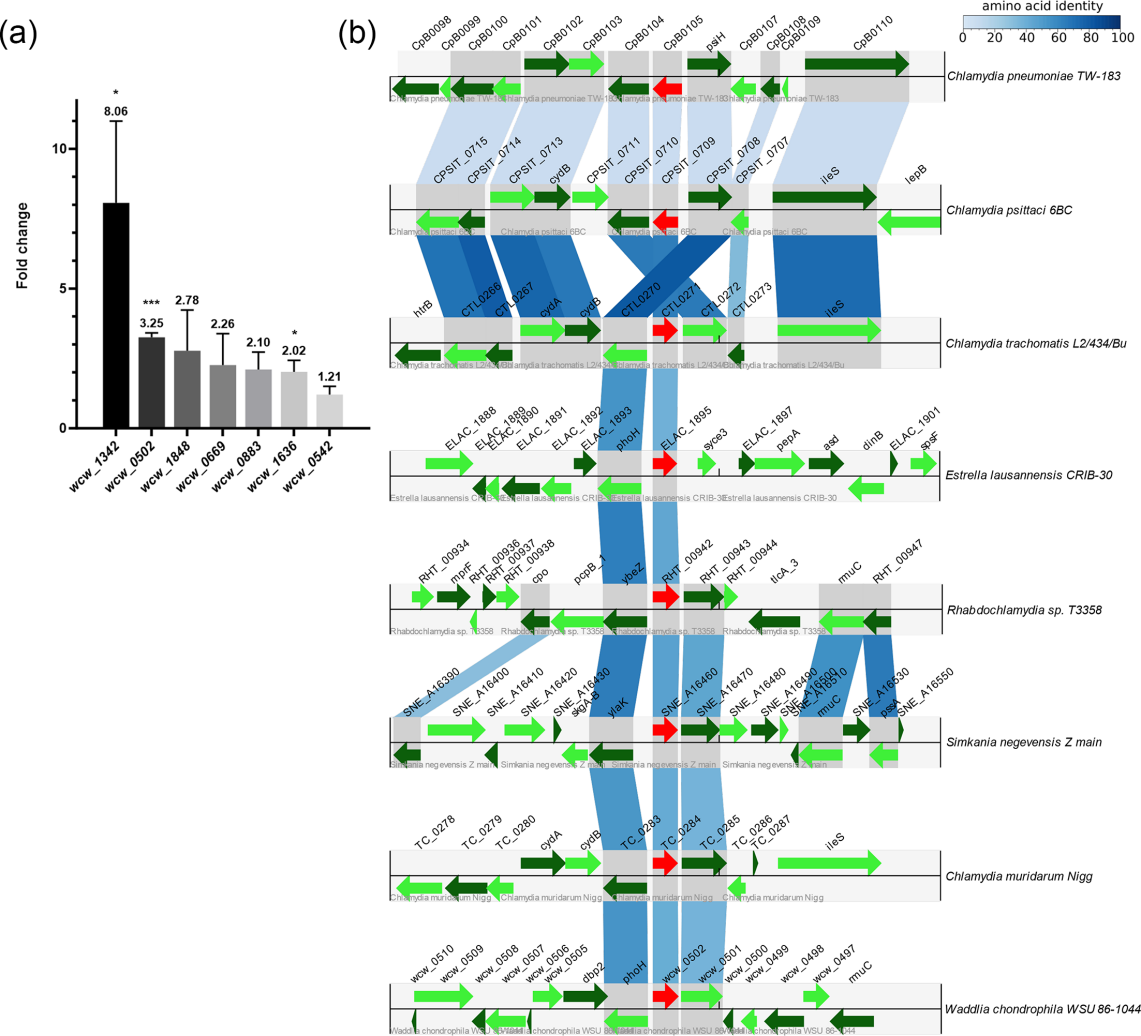
*wcw_0502* is upregulated in *W. chondrophila* ABs induced by iron starvation and has a homologue in every investigated member of the *Chlamydiota* phylum. (a) RT-qPCR confirms upregulation in iron-starved ABs of the seven genes identified in RNA-seq analysis and is listed in Table 2. McCoy cells were infected with *W. chondrophila* and treated or not with 2,2′-bipyridyl 8 hpi. Samples were collected at 24 hpi, and RNA was extracted. qPCR data represent the fold change between treated and untreated samples. Each error bar represents the standard deviation of three biological replicates. Significance is calculated using the unpaired t-test where *P*<0.05 (*), *P*<0.01 (**) and *P*<0.001 (***). (b) *wcw_0501*, *wcw_0502* and *wcw_0503* are encoded together in every examined member of the *Chlamydiota* phylum except for *E. lausannensis*. A region of 8,000 bp around *wcw_0502* and its homologues was drawn using ChlamDB (30), and the conservation of the genes is shown with the blue projection. The darker the blue, the higher the amino acid identity.

The upregulation of selected genes in response to iron starvation was validated by qPCR under the same treatment conditions as used for the RNA-seq experiments (Fig. 1a). Among the seven selected genes, three are linked to stress response based on UniProt annotations and protein domain predictions [35]: By RT-qPCR, *groE1* (*wcw_1342*) and *groES3* (*wcw_1848*) were upregulated 8.06-fold and 2.78-fold in ABs compared to RBs, respectively. These genes encode chaperones that play a critical role in maintaining protein homeostasis during cellular stress by refolding misfolded proteins and maintaining protein function [36,37]. Additionally, *hrcA* (*wcw_1636*), a transcriptional regulator of stress response genes, was upregulated 2.02-fold [38].

The other selected genes were *dnaB* (*wcw_0669*: 2.26-fold upregulation), which is involved in DNA replication by unwinding the double-stranded DNA, *secA* (*wcw_0883*: 2.10-fold upregulation), described as a protein translocator, and *fabG* (*wcw_0542*: 1.21-fold upregulation), involved in fatty acid biosynthesis [39,41]. Finally, *wcw_0502* emerged as the most intriguing candidate. This gene, which showed a 3.25-fold upregulation by RT- qPCR and encodes for an unknown, hypothetical protein, is present as a single copy in all chlamydial species analysed, and it has no homologue in other, non-chlamydial organisms [35].

In most chlamydial genomes across the phylum, *wcw_0502* is co-encoded with *wcw_0501* and *wcw_0503,* or their homologues, in a conserved manner (Fig. 1b). The only observed exception is *Estrella lausannensis* [42] for which the homologue of *wcw_0501* is encoded elsewhere in the genome [43]. With an intergenic region of 109 bp between *wcw_0501* and *wcw_0502*, these two genes are unlikely part of a single operon. Moreover, our results showing different RNA expressions also support the idea that these two genes are independently regulated. The function of Wcw_0501 is unknown, but it is bioinformatically predicted to be secreted by the T3SS [35]. It was also previously shown to be secreted *in vitro* and to be associated with PG [44,45]. Wcw_0503 is composed of a PilT N-terminus (PIN) domain linked to a PhoH domain. This combination is described as PhoH2, a protein found in both archaea and bacteria, with various described roles such as in toxin-antitoxin modules in *Mycobacterium tuberculosis* or phosphate starvation response [46,48].

### *wcw_0501*, *wcw_0502* and *wcw_0503 *are stably expressed during the *W. chondrophila* lifecycle, but the Wcw_0502 protein is not detected in EBs

**Fig. 2.**
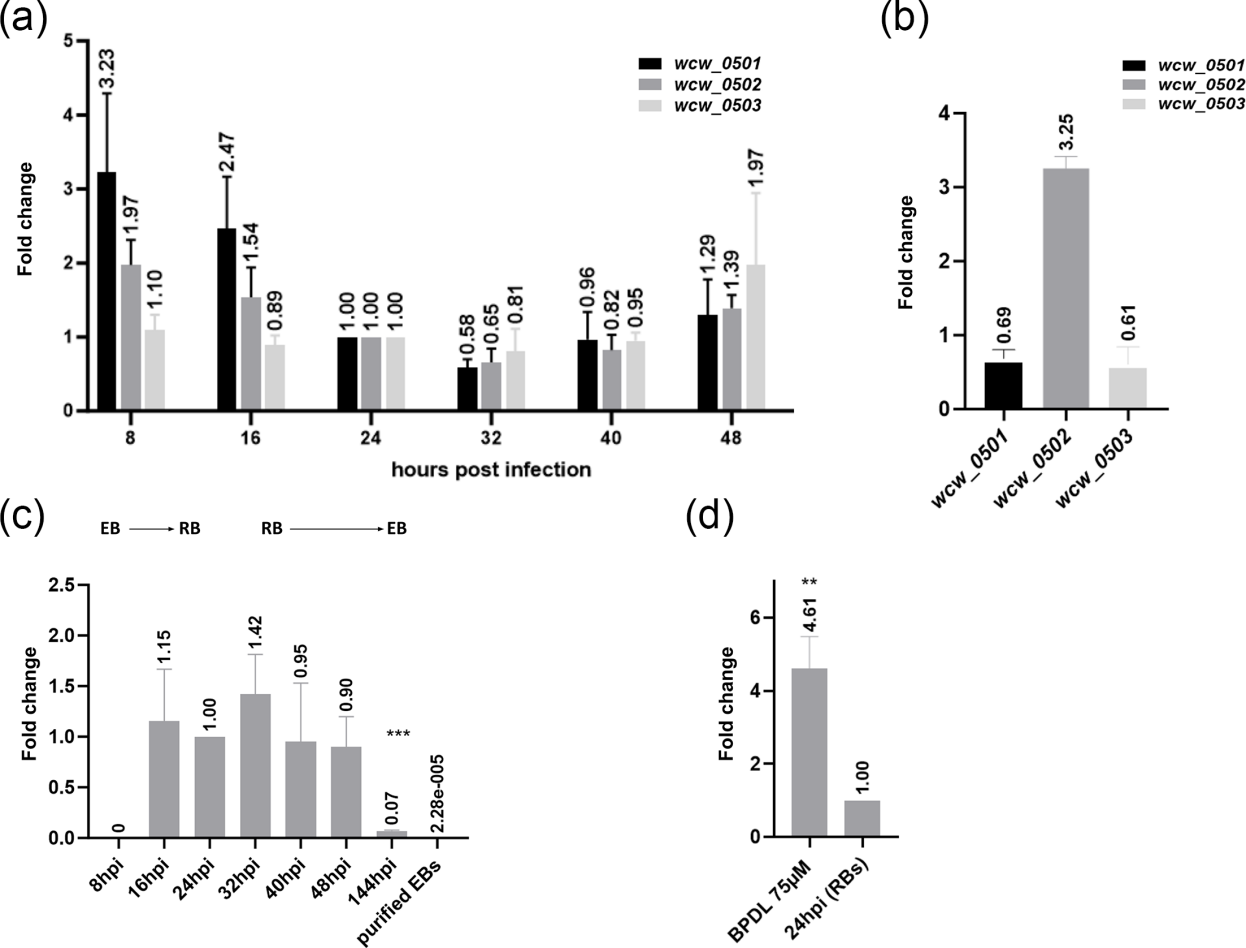
During the life cycle of *W. chondrophila*, *wcw_0501*, *wcw_0502* and *wcw_0503* are stably expressed. *wcw_0502* is upregulated upon iron starvation, both at the mRNA and protein levels. McCoy cells were infected with *W. chondrophila* and collected at different time points during infection (a and c). For aberrant bodies, cells were treated at 8 hpi with 2,2′-bipyridyl and collected at 24 hpi (b and d). RNA was extracted and processed to cDNA before performing qPCR. (a) *wcw_0501*, *wcw_0502* and *wcw_0503* are stably expressed during the bacterial lifecycle (0 to 48 hpi). (b) Compared to untreated control samples, only *wcw_0502* is upregulated in iron-starved ABs. Protein levels were quantified using immunoblot with anti-Wcw_0502 antibodies. Quantification was performed by reporting the signal intensity to the number of genome copies (assessed by qPCR), and fold changes are relative to 24 hpi. (c) Protein expression is higher in RBs (16–32 hpi) than in EBs (144 hpi), and (d) is 4.61 times higher in ABs than in RBs (24 hpi). Each error bar represents the standard deviation of three biological replicates. Significance is calculated using the unpaired t-test where *P*<0.05 (*), *P*<0.01 (**) and *P*<0.001 (***).

To investigate the role of *wcw_0501*, *wcw_0502* and *wcw_0503* in the *W. chondrophila* lifecycle, we first characterized their expression in the absence of stress. RNA and protein levels were analysed following infection of McCoy cells with *W. chondrophila*, from 8 to 48 hpi. Since the comparison between RBs and ABs was done at 24 hpi, all the RT-qPCR results were standardized to the 16S rRNA of the 24 hpi, untreated sample. No statistically significant variation of mRNA levels was observed for the three genes of interest during the lifecycle (Fig. 2a). In ABs induced by iron starvation, mRNA levels of *wcw_0501* and *wcw_0503* were unaffected by the treatment, but *wcw_0502* was significantly upregulated (3.25-fold, *P*<0.001), confirming the results of the RNA-seq analysis (Fig. 2b).

Quantification of the Wcw_0502 protein in infected cells was performed by immunoblot with anti-Wcw_0502 antibodies (Fig. 2c and Data S1). The signal on the immunoblot was normalized to the genome copy number (16S rRNA) at each corresponding timepoint. The intensity of the signals was quantified, and fold change was calculated compared to the 24 hpi untreated sample. At early timepoints, the protein levels were below the detection limit, but from 16 hpi onwards, the Wcw_0502 protein was detected. During the bacterial replication phase (24–32 hpi), Wcw_0502 levels were stable. Following redifferentiation to EBs (40 hpi onwards), the Wcw_0502 level diminished drastically, and the protein was not detected in purified EBs. These results were consistent with the RNA sequencing results detailed previously (Fig. 1a) as well as with RT-qPCR results (Fig. 2b) as Wcw_0502 protein is 4.61-fold more abundant in ABs induced by iron starvation than in untreated RBs (Fig. 2d).

### Wcw_0502 and CTL0271 are secreted by the T3SS of *Y. enterocolitica*

**Fig. 3.**
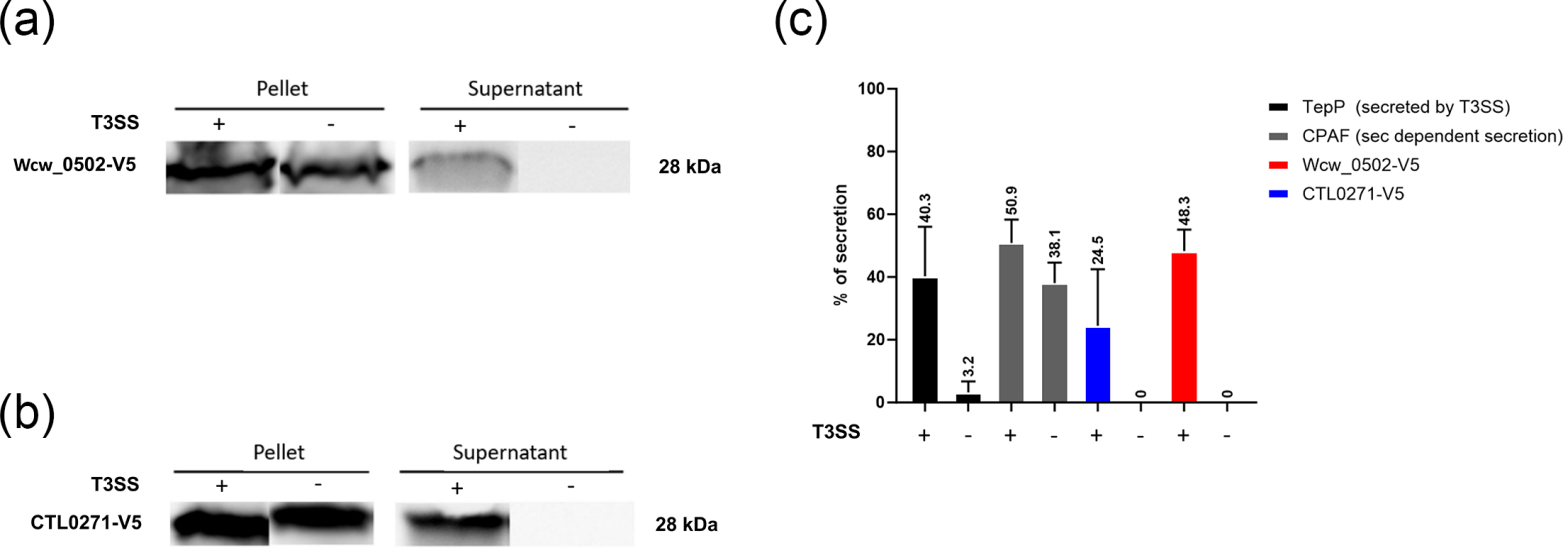
Wcw_0502 and CTL0271 are secreted by the T3SS of *Y. enterocolitica*. Two *Y. enterocolitica* strains were transformed with a plasmid encoding for Wcw_0502-V5 or CTL0271-V5. One strain possesses a functional T3SS, and the other expresses a non-functional T3SS (44). Immunoblot was performed on the culture supernatants and pellets of both *Yersinia* strains expressing Wcw_0502-V5 (a) or CTL0271-V5 (b). The presence of both proteins in the supernatant of the T3SS functional strain coupled to their absence in the supernatant of the T3SS-deficient strain confirms the secretion of Wcw_0502 and CTL0271 by the T3SS. (c) Relative quantification of the secretion of Wcw_0502 and CTL0271 in both *Yersinia* strains compared to CPAF (secreted but not T3SS-dependent) and TepP controls (T3SS effector) [39,40]. Each error bar represents the SD of three biological replicates. Significance is calculated using the unpaired t-test where *P*<0.05 (*), *P*<0.01 (**) and *P*<0.001 (***).

Wcw_0502 is predicted to be a T3SS effector by two out of four algorithms on ChlamDB, our homemade comparative genomics database of the *Chlamydiota* phylum (EffectiveT3 and T3_MM) [35,49, 50]. To confirm this prediction, we used *Y. enterocolitica* as a heterologous expression system [50]. Two strains of *Y. enterocolitica* were used, one with a functional T3SS and one without. A V5-tagged version of Wcw_0502 was expressed in the two strains, and secretion was assessed by immunoblot using an anti-V5 antibody. Wcw_0502 was only detected in the culture supernatant of *Y. enterocolitica* harbouring functional T3SS machinery, indicating T3-dependent secretion (Fig. 3a). Quantification of signal intensity indicated a relative secretion level similar to the one of TepP, a known *C. trachomatis* T3SS effector used as positive control (Fig. 3c) [51]. Interestingly, the same could be observed for CTL0271-V5, the *C. trachomatis* L2 homologue of Wcw*_*0502 (Fig. 3b, c).

To then assess localization of Wcw_0502 during infection, immunofluorescence staining and imaging were performed following *W. chondrophila* infection of McCoy cells. Localization appeared mostly bacterial, with a few weak cytosolic patches. There was no clear host cell interactant (Data S2). To increase the Wcw_0502 signal, HEK 293T cells were transfected with a plasmid encoding for Wcw_0502 with a C-terminal V5 tag. The observed signal was only cytosolic (Data S3). Transfection or transfection with *W. chondrophila* infection was then performed in another cell line, HeLa cells. There, Wcw_0502 was detected in the cytosol and the nucleus (Data S4). Co-staining of transfected HeLa cells with anti-calnexin (endoplasmic reticulum) or mitotracker (mitochondria) did not show any co-localization. Cell cultures were also fractionated to separate the nuclei, heavy membranes and light membranes into fractions to determine whether the protein may be enriched in a particular cellular compartment. Wcw_0502 was observed, by Western blot, in all of these fractions (Data S5). Additionally, the impact of Wcw_0502-V5 transfection on HeLa cells mortality was determined by propidium iodide assay, which enters into dead cells but not live ones. No significant increase in mortality was found compared to the negative controls or when the transfected cells were also infected (Data S9).

### Wcw_0502, CTL0271 and their homologues contain a cystine knot-like domain usually found in eukaryotes

**Fig. 4.**
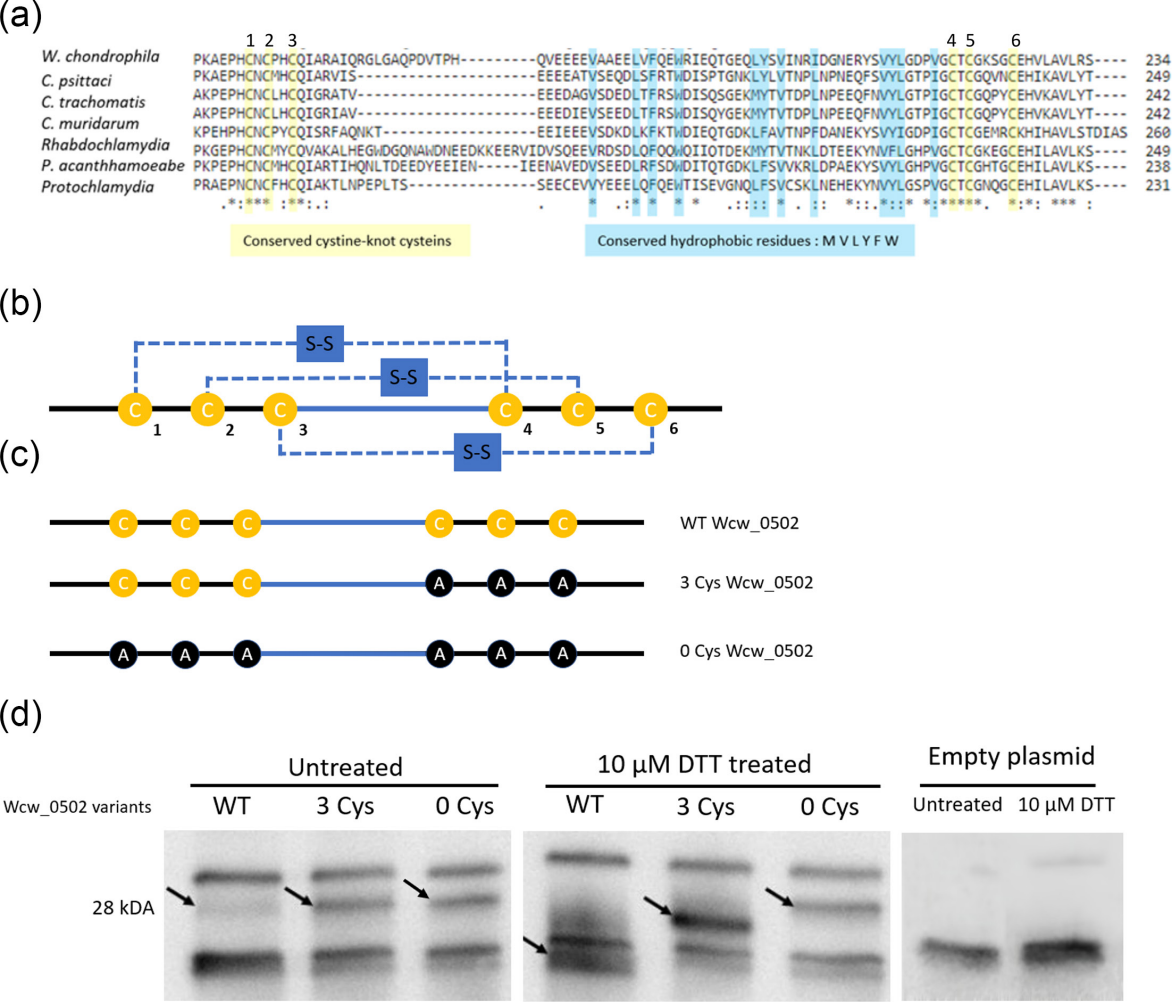
Wcw_0502 displays a cystine knot-like domain that is conserved in all members of the *Chlamydiota* phylum. (a) Alignment of the C-terminal portion of Wcw_0502 and its homologues in different members of the *Chlamydiota* phylum. Six cysteines (yellow) and a region with hydrophobic residues (blue) between C3 and C4 are conserved in all species. (b) Schematic representations of the structure of a cystine knot domain. S-S indicates the disulphide bridges resulting in the knot shape. (c) Schematic of the cystine knot domain in WT Wcw_0502 and in the two mutants. Either 3 (3 Cys) or all (0 Cys) cysteine residues were substituted with alanine. (d) Immunoblot of WT Wcw_0502 and 3 Cys and 0 Cys variants expressed by an IPTG inducible promoter in *E. coli*. Samples were treated or not with 10 μM DTT. Black arrows point at the bands corresponding to Wcw_0502.

Phyre2 was used to identify domains present in Wcw_0502 [52]. Interestingly, a cystine knot domain in the C-terminal region of the protein was partially identified. Upon inspection of the amino acid sequence, six cysteines (positions 108 (C1), 110 (C2), 113 (C3), 153 (C4), 155 (C5) and 161 (C6) in *W. chondrophila* Wcw_0502) were found. These residues are conserved in Wcw_0502 homologues of other *Chlamydiota* members. A highly conserved patch of hydrophobic residues between C3 and C4 might promote hydrophobic interactions (Fig. 4a). Taken together, these features resemble a C-terminal cystine-knot structure [53]. Cystine knots are stable protein structural motifs characterized by disulphide bonds that form a knot structure, protecting the protein from chemical stress. They are commonly found in secreted peptides and proteins such as toxins, growth factors and hormones but have been exclusively described in eukaryotic organisms [53,56].

To determine if the cysteines in Wcw_0502 can form disulphide bonds, we expressed WT Wcw_0502, as well as two mutated variants (three Cys, with C4, C5 and C6 substituted with alanine residues; 0 Cys, with all six Cys replaced with alanine residues), in *E. coli* under control of an inducible promoter (Fig. 4c). Lysates of these cultures were then treated or not with a reducing agent, DTT. Untreated Wcw_0502 variants migrated to the expected protein size (28 kDa). When treated with DTT, the variant without Cys residues migrated like the untreated samples, but the three Cys variant and WT protein exhibited a shift in the migration. They both migrated faster than in the untreated samples, indicating that DTT influenced the cysteines present in that region (Fig. 4d). While we are not able to explain the mechanism of this shift towards a smaller molecular weight, it appears linked to the action of DTT on these specific cysteine residues.

As Wcw_0502 is secreted by the T3SS and has a putative domain associated with protein–protein interactions, the identification of a potential target of Wcw_0502 in host cells was attempted using co-immunoprecipitation (Co-IP) from HEK 293T cells expressing Wcw_0502 with a C-terminal V5 tag. However, the MS/MS analysis of the Co-IP sample led to weak signals, and no clear candidate could be identified. Although some proteins that bind DNA were identified in the experiment, this result does not coordinate with the results from the immunofluorescence experiment on these same transfected cells, as these indicated a cytosolic localization for Wcw_0502 (Data S3 and S4).

### The expression of *wcw_0502* and *ctl0271* depends on the type of stress applied on infected cells

**Fig. 5.**
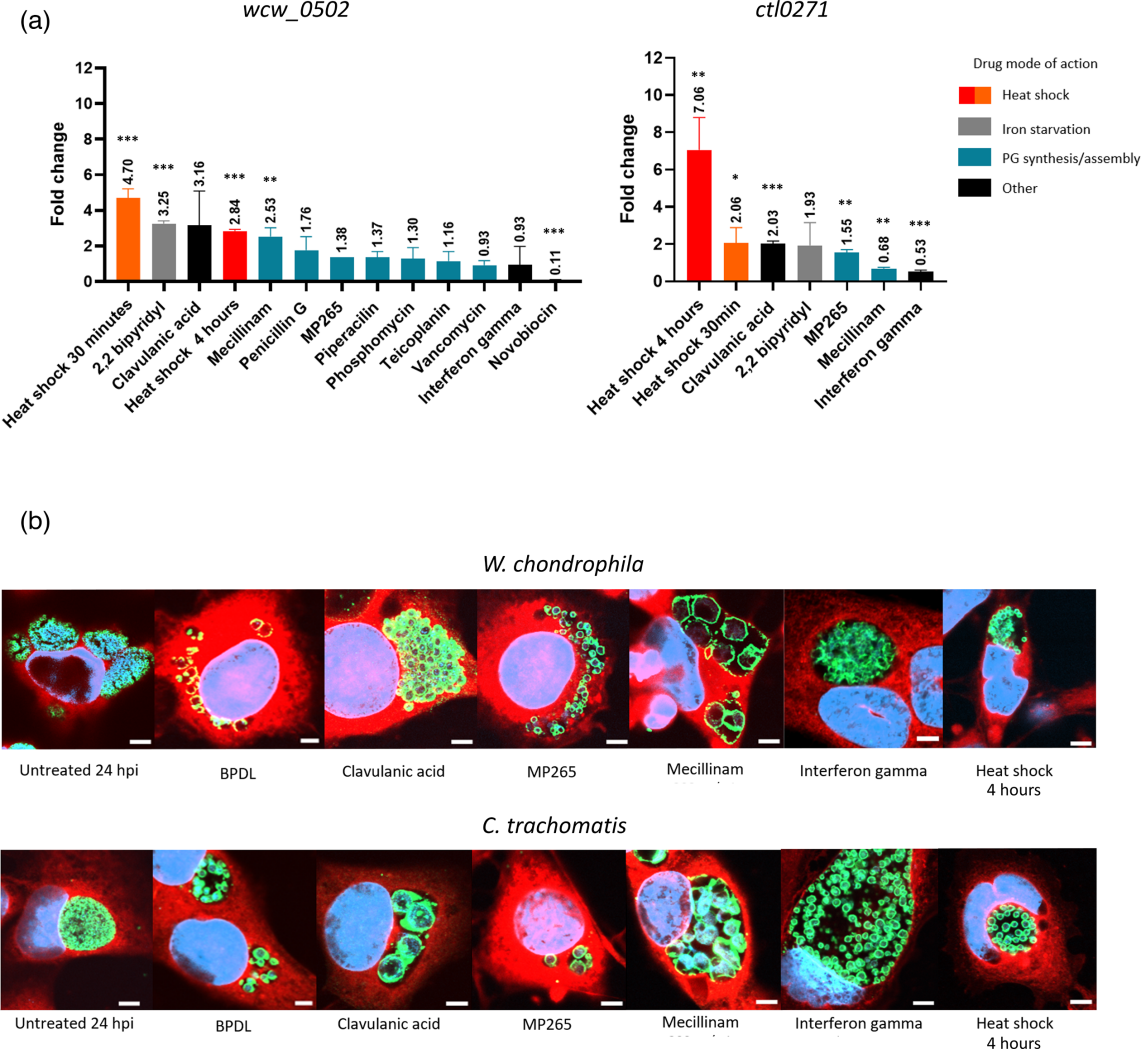
*wcw_0502* upregulation and morphology of *W. chondrophila* are stress specific. McCoy cells (or HeLa cells for IFNγ treatment) were infected with *W. chondrophila* or *C. trachomatis*. From 8 to 24 hpi, the cultures were incubated with or without the stressors listed in Table 1. (a) RNA was extracted from treated and untreated samples, and cDNA was analysed by RT-qPCR. Expression levels of *wcw_0502* (left) or its *C. trachomatis* homologue CTL0271 (right) following treatment of infected cells by different stressors vs. untreated. Each error bar represents the sd of three biological replicates. Significance is calculated using the unpaired t-test where *P*<0.05 (*), *P*<0.01 (**) and *P*<0.001 (***). (b) Immunofluorescence staining of *W. chondrophila* and *C. trachomatis*-infected cells treated with the different AB-inducing stressors. Scale bar, 5 μm. Coloration: blue, DNA (DAPI); red, cytosol (concanavalin A); green, bacterial membrane (*C. trachomatis* MOMP or *W. chondrophila* OMPs).

To quantify the impact of different types of stress (nutrient starvation, PG targeting antibiotics, replication inhibition, heat shock and immune response) on *wcw_0502* expression, *W. chondrophila-*infected cells were treated as in Scherler *et al.* (Fig. 5a) [16]. Additional treatments known to induce AB formation, MP265 (inhibitor of division), 42°C heat shock and IFN gamma (IFNγ) were also included. Of the 13 different stressors applied on *W. chondrophila-*infected cells, only BPDL, heat shock, clavulanic acid and mecillinam induced an upregulation of *wcw_0502* by more than twofold, with BPDL and heat shock exhibiting the strongest upregulations (4.92- and 4.78-fold upregulation compared to untreated control). Interestingly, the upregulation observed after 30 min of heat shock at 42°C (4.70-fold) was stronger than the one observed after 4 h at 42 °C (2.84-fold). Conversely, a significant downregulation of *wcw_0502* was observed following treatment with novobiocin (0.11-fold compared to the untreated control).

Similarly*, ctl0271* was upregulated following heat shock at 42 °C and clavulanic acid treatment. However, contrarily to what was observed for *wcw_0502*, the upregulation by heat shock was stronger after 4 h than after 30 min (7.06- and 2.06-fold, respectively). Iron starvation and mecillinam did not significantly upregulate *ctl0271* expression. Additionally, immunofluorescence imaging of *W. chondrophila-* and *C. trachomatis-*infected cells following treatment showed that the different stressors applied resulted in various AB morphologies (Fig. 5b). The upregulation of *wcw_0502* and *ctl0271*, however, did not correlate with a specific AB morphology.

### The *wcw_0502* and *ctl0271* expression is likely regulated by HrcA

RNA sequencing indicated that *W. chondrophila hrcA* was upregulated during iron starvation, alongside *wcw_0502*, *groES3* and *groE1* (Table 2). In addition, our results demonstrated that *wcw_0502* is also upregulated following heat shock (Fig. 5), as it was previously found for its homologue in *C. trachomatis* (51). HrcA is a transcriptional regulator that controls the expression of a broad range of stress-related genes in bacteria by recognizing a specific DNA regulatory sequence known as the CIRCE sequence [TAGCA-(N_15_)-TGCTAA] in the promoter region of the target genes [57,59]. Stress response genes such as *groES3* and *groE1* are normally repressed by HrcA when it is bound to the CIRCE motif. This sequence is present upstream of several putative stress-related genes in *W. chondrophila*, including the short intergenic region between *wcw_0502* and *wcw_0503* (53). This regulatory sequence could also be observed in the intergenic region between the homologues of *wcw_0502* and *wcw_0503* in other members of the *Chlamydiota* phylum (Data S7). As HrcA is usually a repressor, *wcw_0502*, *groES3* and *groE1* expression should be repressed when HrcA is upregulated. However, our data showed the opposite, as these genes appeared upregulated at the same time as their putative repressor.

**Fig. 6.**
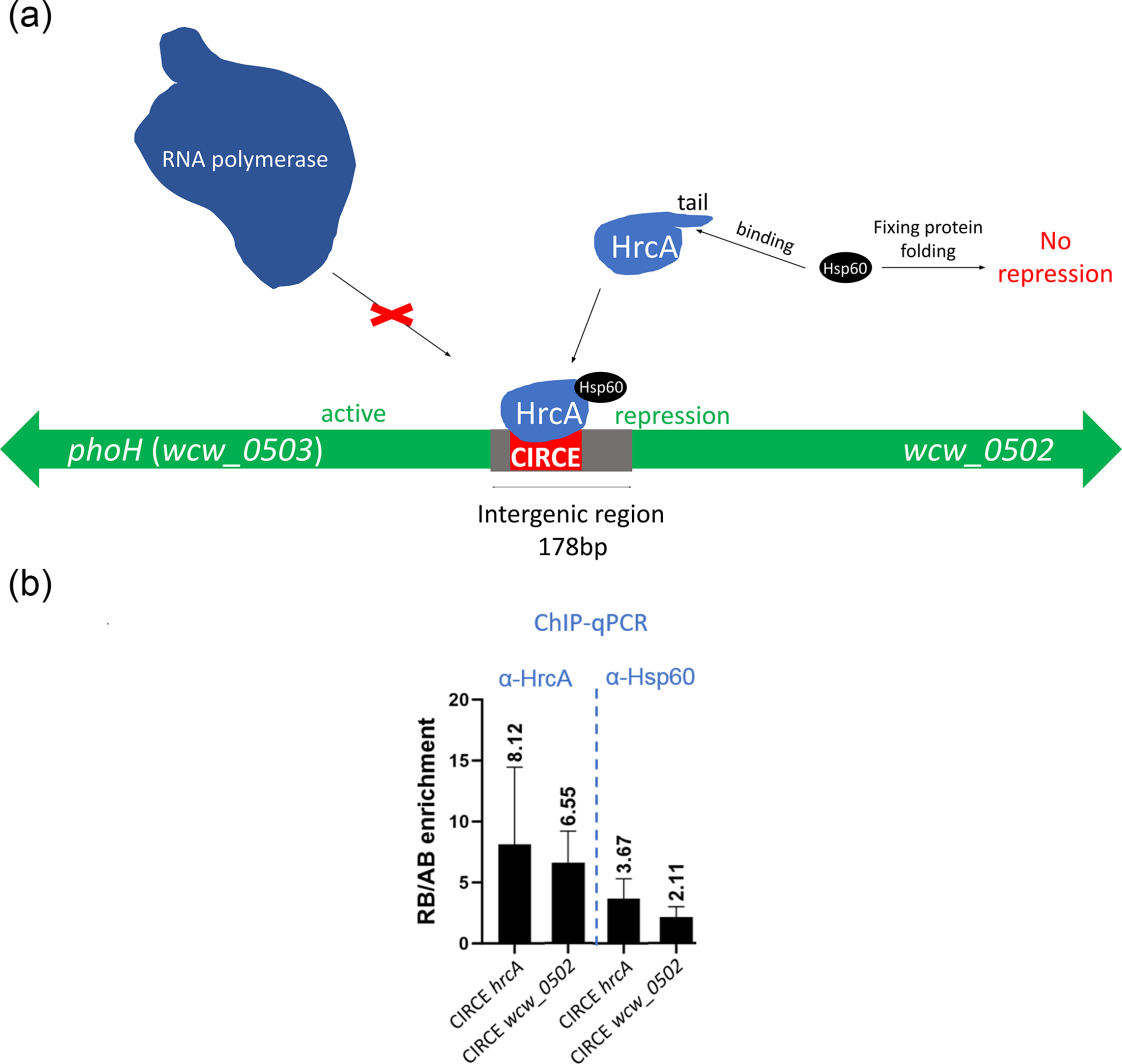
HrcA binds upstream of *wcw_0502* to repress its expression during non-stressful conditions. This repression is alleviated during iron starvation. (a) Scheme representing the regulation of the divergent genes *wcw_0502* and *wcw_0503* by HrcA/Hsp60. The intergenic region between *wcw_0502* and *wcw_0503* contains the CIRCE consensus sequence recognized by HrcA. In *W. chondrophila*, HrcA may require the binding of Hsp60 to its C-terminal tail ito bind CIRCE sequences along the genome, as it was shown for *C. trachomatis* (58). If Hsp60 is not associated with HrcA, the latter may not bind to CIRCE sequences, releasing the repression of downstream genes. (b) Occupancy of HrcA and Hsp60 at the CIRCE sequence upstream of hrcA and *wcw_0502* in RBs compared to iron-starved ABs. Enrichment was assessed by ChIP-qPCR using HrcA or Hsp60 as bait proteins.

Interestingly, an additional level of HrcA regulation has been described in the *Chlamydiaceae* (54): in this case, the C-terminal part of the HrcA protein has an extended tail, which is not present in other bacteria. This tail can bind to Hsp60 (Fig. 6a and Data S8). This association with Hsp60 is required for HrcA to bind to the CIRCE sequences of the target genes. Amino acid sequence analysis of *W. chondrophila* HrcA revealed a similar extended tail, and the same is true for multiple members of the *Chlamydiota* phylum such as *Simkania negevensis*, *E. lausannensis*, *Neochlamydia* or *C. suis* (Data S8).

To clarify whether the upregulation of *wcw_0502*, upon iron starvation or heat shock, is due to the inability of HrcA to bind the CIRCE sequence in the *wcw_0502-wcw_0503* intergenic region, chromatin immunoprecipitation followed by qPCR (ChIP-qPCR) with anti-HrcA antibodies was performed. The relative occupancy of HrcA at the *hrcA* and *wcw_0502* CIRCE sequences was compared between RBs and iron-starved ABs (Fig. 6b). Our results demonstrated that, during iron-starvation, HrcA binds less often to CIRCE sequence upstream of *hrcA* and *wcw_0502*, than in non-stressed RBs, which could explain the observed upregulation of these genes. Additionally, the same experiment was performed using anti-Hsp60 antibodies, which showed that the Hsp-60 occupancy at the CIRCE sequences was also higher in RBs than in ABs. These results suggest that (1) the upregulation of *wcw_0502* upon iron starvation may be due to the inability of HrcA to bind the upstream CIRCE sequence in these conditions and (2) Hsp60 binds indirectly to CIRCE sequences via its interaction with HrcA. Altogether, our results strengthen the above-mentioned hypothesis that Hsp60 binding to HrcA is required for HrcA-mediated repression of stress-related genes (Fig. 6a).

## Discussion

In this study, the gene expression of *W. chondrophila wcw_0502* was characterized during stress response. This gene is significantly upregulated in ABs induced by iron starvation and heat shock but not during disruption of PG synthesis or PG assembly (Fig. 5a). Interestingly, Wcw_0502 is conserved in all known members of the *Chlamydiota* phylum, despite the broad genetic variability between these organisms, which suggests a potentially important role for this hypothetical protein in chlamydial biology. Furthermore, the genomic region surrounding *wcw_0502* (including *wcw_0501* and *wcw_0503*) is also conserved, hinting at a co-regulation of these three genes (Fig. 1b).In other species of the *Chlamydiota* phylum, *wcw_0502* homologues are not upregulated during iron starvation (Data S9). This result suggests that mechanisms underlying persistence are not shared between distant chlamydial species. However, following heat shock, a similar regulation of *wcw_0502* and *ctl0271*, its *C. trachomatis* homologue, was observed. This indicates a likely conserved regulation mechanism in these conditions*.* Upregulation of *wcw_0502* was shown to be stress-specific since it has been observed mostly during heat-shock and iron deprivation and not during PG-related stress*.*

Wcw_0502 is a predicted T3SS effector and was shown here to be secreted by the T3SS in the *Y. enterocolitica* heterologous system [50]. Similar observations were made with its *C. trachomatis* homologue CTL0271 (from the L2 serovar), indicating that T3-dependent secretion could be a conserved feature in all Wcw_0502 homologues (Fig. 3c). However, immunofluorescence staining of Wcw_0502 during infection could not confirm secretion, since the protein signal co-localized with bacteria (Data S2). This may be due to the low sensitivity of this assay, as maybe only a small proportion of the protein pool is secreted at any given time [60]. However, as this putative T3SS effector is present in every known member of the *Chlamydiota* phylum, and as the protein was only detected in RBs, we can hypothesize that it may serve an important function during the chlamydial replication cycle, a function that seemingly does not impact the mortality rate of the host cell. A recent pre-print article studied putative T3-secreted effectors in *C. trachomatis* L2 expressing multiple such effectors fused to different tags for secretion assessment [61]. The homologue of Wcw_0502 (CT_016 in the *C. trachomatis* D serovar) was one of the tested candidates, and its secretion could be confirmed by two out of three of the assays performed, which strongly supports the fact that CT_016 is secreted in *C. trachomatis* [61]. Combined with our *Y. enterocolitica* T3 secretion assay, the hypothesis that Wcw_0502 represents a rare case of T3 secretion effector conserved in the entire *Chlamydiota* phylum is strengthened. To date, only two T3 effectors potentially share such conservation: CopN and Pkn5 [3]. In the previously mentioned secretion study, the detection of CT_016 in transfected HeLa cells by immunofluorescence also showed a cytosolic signal, similar to what we observed with Wcw_0502 (Data S3 and S4).

Phyre2 identified a cystine knot domain in the C-terminal region of Wcw_502, CTL0271 and their homologues [52]. Cystine knots have been reported in various secreted proteins from plants, animals, insects and fungi [53]. However, this motif was never described in a bacterial protein. Cystine knots have been identified in proteins with a wide range of functions, including toxins, defensins, growth factors, mucins and more [42,45]. This structural domain can confer high resistance to proteolytic, thermic and chemical stressors. Additionally, the disulphide bonds create loops that expose peptides on the outside of the protein. These peptides are often composed of hydrophobic residues, as in Wcw_0502 and its homologues, or contain an additional cysteine, which favors the formation of a disulphide bond with other proteins in the extracellular matrix [53,55] (Fig. 4a).

DTT treatment of WT Wcw_502 and its mutated versions resulted in a shift in the migration of the proteins detected by immunoblot, which supports the existence of disulfide bonds between the six cysteines. This observation is compatible with a cystine knot structure where disulphide bonds normally occur between cysteine residues 1 and 4, 2 and 5 and 3 and 6 (Fig. 4b). A similar migration shift was observed with the rat mucin protein Muc2, which possesses a C-terminal cystine-knot used as a positive control [55]. Muc2 polymerizes into mucin and the monomer migrated faster when the sample was treated with DTT, similarly to what we observed with Wcw_0502. We were not able to determine precisely which cysteine residues are implicated in the disulphide bonds. However, the migration shift observed when the Wcw_0502 variant lacking the last three cysteines was treated with DTT indicates that a disulfide bond was present in this protein, even though cysteines 1, 2 and 3 do not usually make disulphide bonds between them (Fig. 4d). It is unclear whether this bond also occurs in the WT protein or if it is an artefact due to the absence of the three last cysteine residues. Additionally, when produced in *E. coli*, the Wcw_0502 signal on the Western blot was weaker than for the mutants having cysteine swapped to alanines in the cystine-knot region (Fig. 4d). It is unknown whether this occurs (i) due to an increased pressure for *E. coli* to produce the non-mutated protein or (ii) due to a change in affinity of the antibody to its antigen.

In the absence of stress, *wcw_0502* is repressed by HrcA, a conserved stress regulator, and this repression is released when * W. chondrophila-*infected cells are iron-starved or heat-shocked. In a stressful environment, Hsp60 is a chaperone, refolding and repairing proteins [62,63]. It was hypothesized that Hsp60 does not bind to HrcA during stress because the available pool of this chaperone is mobilized elsewhere to execute its protein repair activity (59). If Hsp60 does not bind to HrcA, repression of stress response genes ceases (Fig. 6a, b) Our data support this hypothesis as *wcw_0502* expression increases upon iron starvation and heat shock, two conditions which lead to protein misfolding. It was previously shown that *Caulobacter crescentus*, another Gram-negative bacteria, accumulated hydrogen peroxide, a reactive oxygen species, upon iron deprivation [64]. The accumulation of hydrogen peroxide leads to oxidative stress, which is known to impact protein stability [65]. A similar event might also occur in members of the *Chlamydiota* phylum. We can thus hypothesize a potential link between Wcw_0502 and iron regulators such as YtgR, which also regulates the tryptophan *trp* operon [66]. While this link might not directly involve iron homeostasis, it might indirectly regulate Wcw_0502 solely by keeping iron at levels that avoid oxidative stress.

However, other stressful conditions, such as antibiotic or IFNγ treatment, which induce aberrant body formation without impacting protein folding, do not alleviate the HrcA-dependent repression of *wcw_0502* as was seen here by a lack of upregulation under these conditions (Fig. 5). It was previously reported that *C. trachomatis* has three copies of *hsp60*, but that only one, *groEL1* (*ctl0365*), binds HrcA [67,68]. *W. chondrophila* also has three copies of *hsp60*, and the paralog of *C. trachomatis ctl0365* corresponds to *wcw_1343.* The anti-Hsp60 used in our Chip-qPCR experiment was raised against the paralog of GroEL1 in * S. negevensis* (SNE_a07850, 71% amino acid identity with Wcw_1343), which reduces the probability of cross-reaction events with the other paralogs of *W. chondrophila* Hsp60 (56% and 51% amino-acid identity with the Wcw_1849 *and* Wcw_1396, respectively).

The CIRCE sequence recognized by HrcA is conserved in the intergenic region of all homologues of *wcw_0502* and *wcw_0503*, suggesting that *wcw_0503* may also be regulated by HrcA. Surprisingly, *wcw_0503* was not upregulated following treatment with BPDL, which implies that there may be additional layers of regulation [69,70]. One potential extra layer of regulation was detailed in the study by Raulston *et al.* (54). There, a distant homologue of the *E. coli* ferric uptake regulator (*fur)* was shown to be present in *C. trachomatis* (*ctl0548*). Fur is also a transcriptional repressor. It recognizes a specific DNA sequence (called Fur box) in the regulatory region of genes linked to iron acquisition. Fur activity is regulated by iron levels as it requires binding of [Fe^2+^] or other divalent cations to repress transcription [69,71]. A binding region for Fur was identified at the 5′ region of the coding regions of *ctl0270*, the homologue of *wcw_0503* [72]. However, no close homologue of *ctl0548* could be identified in *W. chondrophila*, although bioinformatic predictions of the functional domain suggest that Wcw_1697 may be a ferric uptake regulator [35]. The observed downregulation of *wcw_0503* during iron starvation could be due to the repression by Fur analog from the binding of another divalent cation such as Mn^2+^ [71,73]. It would be beneficial for future studies to determine whether Wcw_1697 could bind to the putative Fur box sequence in the coding region of *wcw_0503.*

Wcw_0503 and its homologues across the *Chlamydiota* phylum share a common structure composed of a N-terminal PIN domain (an RNA-nuclease) and a C-terminal PhoH domain (together forming PIN-PhoH). This structure has been reported mainly in mycobacteria, associated with another gene as the genomic sequence of *PIN-PhoH* overlaps with the coding sequence of *phoAT* [46,48]. Together, these genes are thought to form a toxin-antitoxin module [46]. Several toxin-antitoxin modules have been uncovered in different members of the *Chlamydiota* phylum [74]. However, in the *Chlamydiota* phylum, no *phoAT* sequence was found. Interestingly, during infection of the cyanobacterium *Synechococcus* spp. by the phage Syn9, the bacterial PIN-PhoH protein, which is also not associated with a phoAT sequence, was found to reduce phage DNA replication and progeny production [75]*.* Another interesting example is *Corynebacterium glutamicum*, where the PIN-PhoH gene is linked to the phosphate starvation response and is upregulated in phosphate deprivation (65). Altogether, proteins containing a PIN-PhoH structure seem to be involved in stress response, whether in phage infection or phosphate deprivation. If expression of *wcw_0503* is induced during phosphate starvation, it could indicate that *wcw_0502* and *wcw_0503* together play a role in protein, iron and phosphate homeostasis.

This study has shown that the expression of *W. chondrophila wcw_0501*, *wcw_0502* and *wcw_0503* is differentially regulated following treatment of infected cells with different types of stressors, which suggests that these highly conserved genes are likely to play an important role in chlamydial stress response. Our results indicate that *wcw_0502* and its *C. trachomatis* homologue *ctl0271* are upregulated in response to conditions that cause protein misfolding. In these conditions, the Hsp60 pool is sequestered, alleviating the repression of stress response genes by HrcA. It is not clear why *ctl0271* is not upregulated during iron starvation*,* but this may be due to alternative regulation mechanisms. Additionally, chlamydia-host compatibility likely defines the bacterial sensitivity to stress and, thus, also the sensitivity of the stress response triggers. As intracellular organisms are known for their highly reduced genomes, it is expectable that the molecular toolbox a species harbours is optimized for the development and survival in a precise host. Infecting another host might limit the efficiency of such a toolbox and lead to more sensitive stress response triggers. Altogether, we think that Wcw_0502 and its homologous proteins are important T3SS effector proteins that, due to their cystine knot-like structure, can operate despite the various stressful proteolytic, chemical and thermic challenges that can occur during infection.

## Supplementary material

10.1099/mic.0.001545 Uncited Supplementary Material 1.

10.1099/mic.0.001545 Uncited Supplementary Material 2.

## Abbreviations

ABs: aberrant bodies
CIRCE: Controlling Inverted Repeat of Chaperone Expression
Co-IP: co-immunoprecipitation
CRB: *Chlamydia*-related bacteria
DMEM: Dulbecco’s Modified Minimal Essential Medium
EBs: elementary bodies
hpi: hours post-infection
IFNγ: IFN gamma
MOMP: major outer membrane protein
PG: peptidoglycan
PIN: PilT N-terminus
qPCR: quantitative PCR
RBs: reticulate bodies
RT: room temperature
TBS: Tris-buffered saline with 0.05% Tween 20
T3SS: type III secretion system
