# Supplementary material for: A conserved Chlamydiota-specific Type III Secretion System effector linked to stress response
Source: Microbiology (Reading). 2025 Apr 28;171(4):001545. doi: 10.1099/mic.0.001545 (PMC12038028; doi:10.1099/mic.0.001545)
Supplement: Uncited Supplementary Material 1. [file mic-171-01545-s001.pdf]

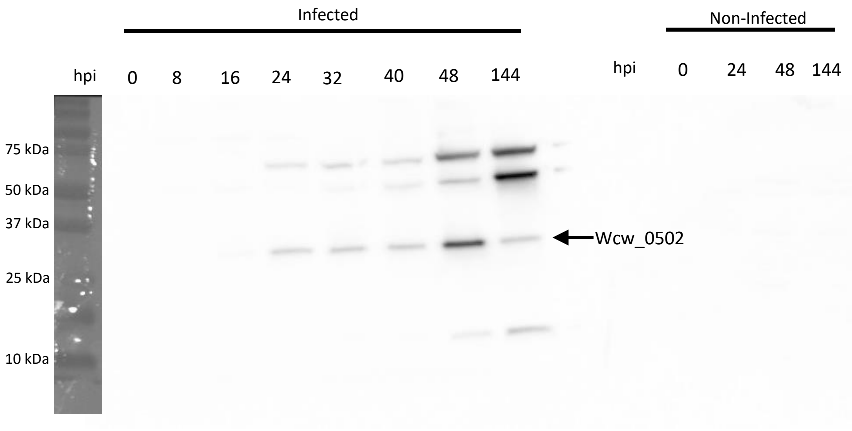

Supplementary Data 1: Representative immunoblot showing Wcw_0502 signal at various time points during *W. chondrophila* infection in McCoy cells, as well as treated or not with BPDL.

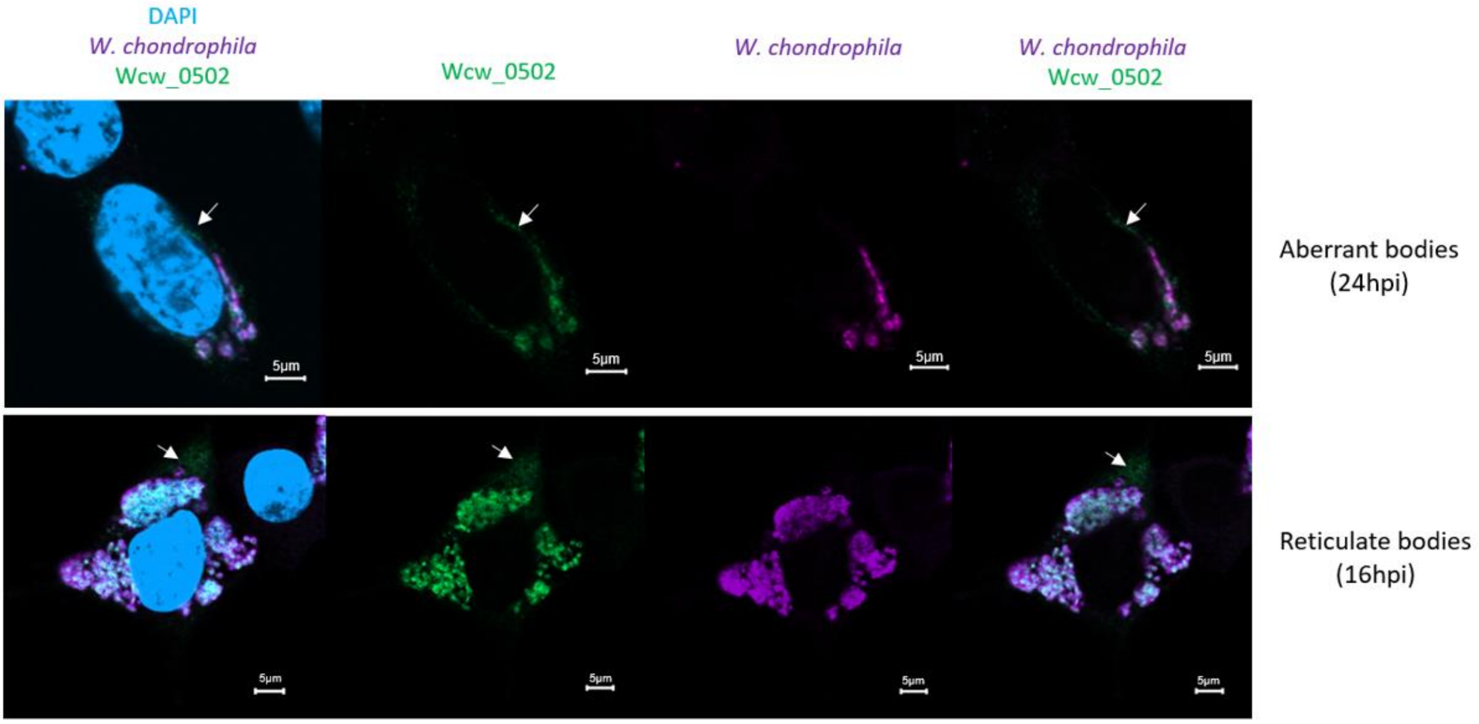

Supplementary Data 2: Wcw_0502 immunofluorescence signal colocalizes with bacteria: Immunofluorescence images of *W. chondrophila*-infected McCoy cells, 24 hpi for ABs or 16 hpi for RBs. ABs were obtained by treatment of infected cells with BPDL 8 hpi. Blue signal represents DNA, purple signal represents *W. chondrophila* and green signal represents the Wcw_0502 protein. White arrows indicates areas of Wcw_0502 protein- within the host cell cytosol.

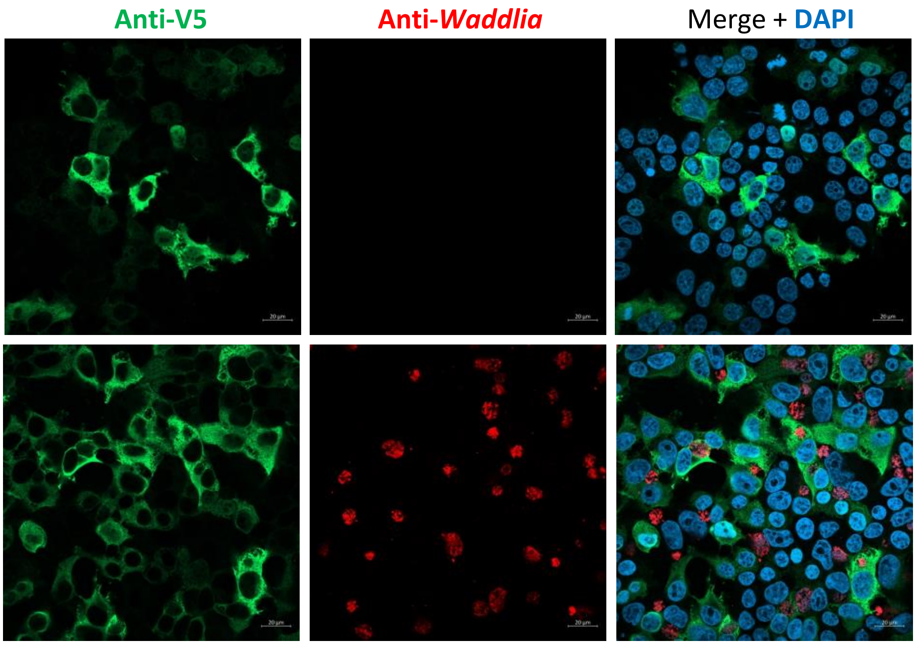

Supplementary Data 3: Wcw_0502-V5 localizes in the host cell cytosol when expressed in HEK 293T cells. Immunofluorescence images displaying HEK 293T cells transfected with a plasmid encoding for Wcw_0502 with a C-terminal V5 tag. Cells were infected (upper panel) or not (lower panel) with *W. chondrophila* 24h post transfection. Cells were fixed 40 hours post transfection. Scale bar : 20 µm

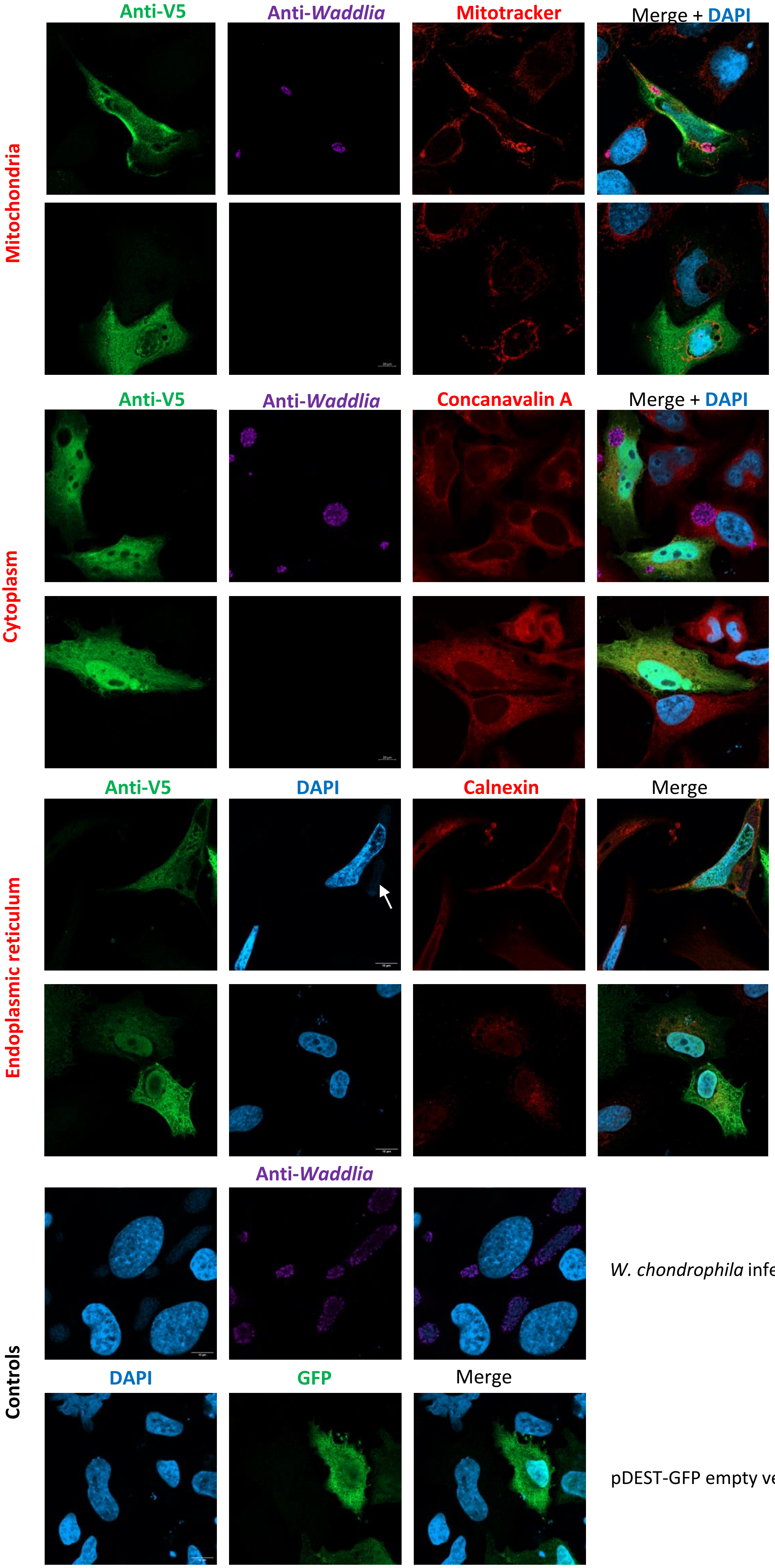

Supplementary Data 4: Wcw_0502-V5 localizes in the host cell cytosol and nucleus when expressed in HeLa cells. Immunofluorescence images displaying HeLa cells transfected with a plasmid encoding for Wcw_0502 with a C-terminal V5 tag. Co-localization with host cell mitochondria, cytoplasm or endoplasmic reticulum (ER) was assessd. Cells were infected (upper panel) or not (lower panel) with *W. chondrophila* 24h post transfection. Cells were fixed 40 hours post transfection. For the ER colocalization experiment, inclusion is designated with the white arrow. There the presence of *W. chondrophil*a is assessed using the DAPI signal. Scale bar : 20 µm

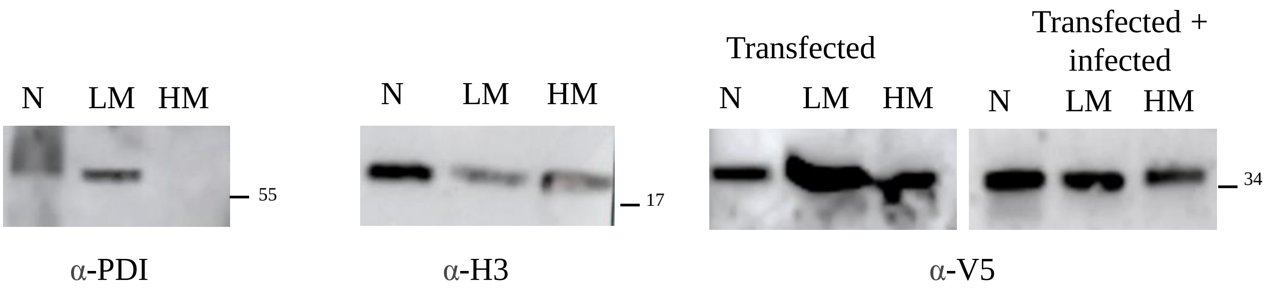

Supplementary Data 5: Western blot on fractionated samples of HeLa cells transfected with pDEST47-*wcw_0502-V5* and infected or not with *W. chondrophila* shows presence of Wcw_0502-V5 in all fractions. N: Nuclear fraction LM: Light membrane fraction HM: Heavy membrane fraction. a-PDI is a LM fraction control and a-H3 histone is a nuclear fraction control.

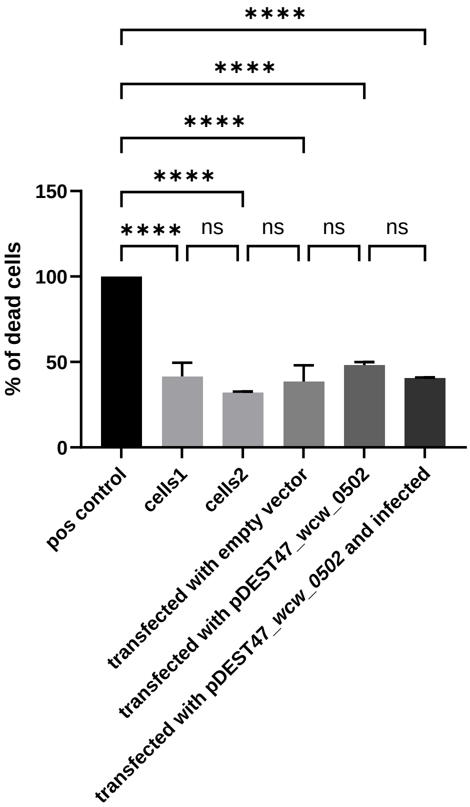

Supplementary Data 6: Wcw_0502-V5 transfection and infection with *W. chondrophila* do not impact cell viability Percentage of dead cells determined by propidium iodide (PI) staining. The positive control (cells treated with Triton X100) shows a significantly higher percentage of dead cells compared to all other conditions. HeLa cells transfected with the pDEST47 vector containing *wcw_0502-V5*, either alone or in combination with *Waddlia chondrophila* infection (24hpi), exhibit no significant difference in cell death compared to cells transfected with an empty vector or non-transfected cells (cells1 and cells2). Each error bar represents the SD of three biological replicates. Significance is calculated using a one-way ANOVA test, were **** indicates $p < 0.0001$.

>*W. chondrophila*

CATACGCTCCTTTTTAATAGCACTTATAGCTTGAGAGTGCTAAATCAATTGACTTAAATAGGTTTATTTAGATATATTTAGTGATTGAATTCAAGAGGTAATTCCAAAGCTCCACATCAACGGAAAAATGAAACGGAGCGTAGAAAAACTCGGTATAAAAAGCAACGT
TTTGAGGTAGATAATG

>C. trachomatis L2/434/Bu(i) 339887 3393675

CATTACAAATTCCTAAATGACTCAAGTGTAAGGGGGAGATAGTACTTTGATTGTGTATCATATCCAGAAAAATTAAAACATGTCTTTGTTAGAGAGAAGTCGGGAGAGAGGGTTTTTAGCAATCAACCTCCGCGTGTGCTAATCTGTTTGTCAAAAATGTACCCCTTA
ACTACAATGCCGAGGAAAGCGAGTCCTTCTGTTGGAGGTTGTTATG

>E. Lausannensis strain CRIB30 1961548 1961928

CACGTTAGGTCTCTGATTTTAATATGGTTGAGTATGTTTGCTAGTAGGAATGTGAAATCCCGGCATCCCGCCCTAATTTTTCTTTTCCGCTGGGTATACCGACAGAGTCTTAAAATTGGGTAGCGGGGCTGTAAGAGGCTGTCCACGAACTCAAATTTTGAGACACTTT
CGGCATACTCAATAAACGGGATAATAAGGTATTTGGGCAAGGGGGAAAAATAGATGTGGAAGTAGCAGTCTAGTGATTCGAGTGCTAAATCAGTTGAAGGCTATTCCCTTTTTTAGTATAATAGGGATATCAGTCAAGCTAGCGGTTAGCTAAAGGCCAGGTTGAC
TATCATGTCTTTAGCTGTCTTGCATACAACCTCCCGGAGGTCTCGCGATG

>Candidatus Rhabdochlamydia sp. T3358 563222 563017

CACTTGATTTCTCCTTTAGTTCAGGTTAAAGTGCAGCATAGCGTGCTCTTTTACTAGACTCAACCTAATTTTTTTAACTAAATTTTAAGTATTTTAACATTAATCTCTTATGAAGTAATTTCAGCAATTGACTATTTCGAGTGCTAAATTCTTGACGCTGAACTTGAAGATC
ACTTATAATGGATTTTGTACTTGAAGGAGAAATTTATG

>P. acanthamoeabe Bn9 isolate 1717807 1718039

CACTAGAACCTCCTTTACCGAACTTTTCAAAGGGGACGCAATTTAGTATGGTGAACCTATGAGCCGAAGCTTACGAAAGAAAGAAAAAAAGTGCGAAACAAAAATGCATGTTGAGAGATCTATTTTTGAACAATTCAGCAATCAACCCCATCGACTGCTAATTTAGTT
TGACGAGAAATCCCCTCTCGAGCTAAGATTGAAACTTCAAGTAGATAAAGAAGTTAAAGGAGCTTTATG

>Protochlamydia W-9 390234 390585

CATTTTTTTTACTCCTAAAAATTAAAAGTGCATCATATTCAGCTCTTTGATCTGAGACAAGAAAAAAATTTAATATTTATTAACCCTAGTTAAATTTTAAGCACTCAAACACTTCAACTGCTAACTTATTGTCAAATATTCTGATTTTGTGTATGATAAGTAAAAATATCAA
AGGGATTAAAAACCCCATATATTGTGATAAAAATTTAATGATTTAAATTTTAAACTAAAAAAAACTTTTTATTACATGATATTAATCTGGATTAGAGGAGGTTGCTCATCATG

>C. muridarum Nigg 339587 339793

CATTACAAGTTCCTAATTCACTCGATGGTAAGAGGTAAACTACTTCGATGTGTATCATATTAATAAAAATTAAAACATGTCTTTATTAGATAGGGAAAACAAGATTGGTTTTTAGCAATCAGGTCGCATGTCTGCTAATCTGTTTGTCAAAAATATCCCACCTTAACTAC
AATGCCGAGGAAAGCGAGTCCTTCTGTTGGAGGTTGTTATG

>C. psittaci WC 752565 752220

CATTCAGCTAACTCCTAACGAAATGGGCTCCAACCCTAATCAAATCATTTTATAACGACAAATATCTTTATTAGTAGAACGATGAAAATACTTAAACAAATACCTGGAGAATCTGCGCCGATGTTGACAAAAATTTATTAGAAACTTCTTGACCCTTTTATATAATTCTA
AGTAAAAAAACTCTATTCTTAATTCTTGCAAAAAGAAAAAACTTCCTGAATCTTTAGGAAGTTAGGTCATGAATTAGCAATCGATATTACGAATTGCTAATTCGATTGTAAAAAAACAAAGCTTCCCTACAATGACGTTAAGCAATGACAGTAGAGTTCACCATTGGAG
GTCGTCATG

Supplementary Data 7: CIRCE Motif in the Intergenic Region of the Wcw_0502 and PhoH Homologs Across Multiple Chlamydial Species. The CIRCE motif (TAGCA-(N)15-TGCTAA) as described by de Barsy (52) is highlighted in the intergenic region between the homologs of the *wcw_0502* and *phoH* genes across multiple *Chlamydia* species, with the conserved TAGCA and TGCTAA sequences in yellow, the variable (N)15 region in green, and mismatches in red.

```
Species             HrcA C-terminal amino-acid sequence                Size
                                                          *:  :
C_trachomatis       RLKVILTQSFYKFKLSFRRPCPTDPRCSQRPAELTRSSSIKLLPAKELS---  389 aa
C_suis              RLKTILTQSFYKFKLSFRKPCPTDSRSSQRPAELTRRSSIKLLPAKELS---  392 aa
W_chondrophila      NISEALTRNIYKFKIQYRQPEPEASYLQKEEHSLLGQSRLMLLENKSKNEAT  392 aa
Neochlamydia        NISEAITRSIYKFKITFRQPQRGTISTQPDKPCFLGESSLMLIEHNGT----  391 aa
S_negevensis        ILSEMLTRNLYKYKITYRQPKTREIGLQTDSTVYLDQAQHLLLEDKR-----  377 aa
E_lausannensis      MISKTLTDAIYKYKITYRQPDAGLPVLFNDKIELNMSKAPILLEDKTFQTP-  397 aa
A_tumefaciens       IMARLSRKQR------------------------------------------  343 aa
M_japonicum         LISRMLR---------------------------------------------  347 aa
B_subtilis          DLSKALTSLYDE----------------------------------------  360 aa
L_lactis            VLTMKLSDYYRYLDGNHYEISK------------------------------  347 aa
                     :
```

Supplementary Data 8: Amino acid alignment of HrcA C-terminal region. When compared to non-related bacteria, HrcA of *W. chondrophila* and other members of the *Chlamydiota* phylum exhibits the same extended tail as HrcA of *C. trachomatis*, suggesting that they also have the ability to bind Hsp60. The conserved residues within the extended area are indicated with red symbols. Red asterisk indicates conservation in all species examined while a red colon indicates high conservation. Alignment was performed with the clustalw web application.

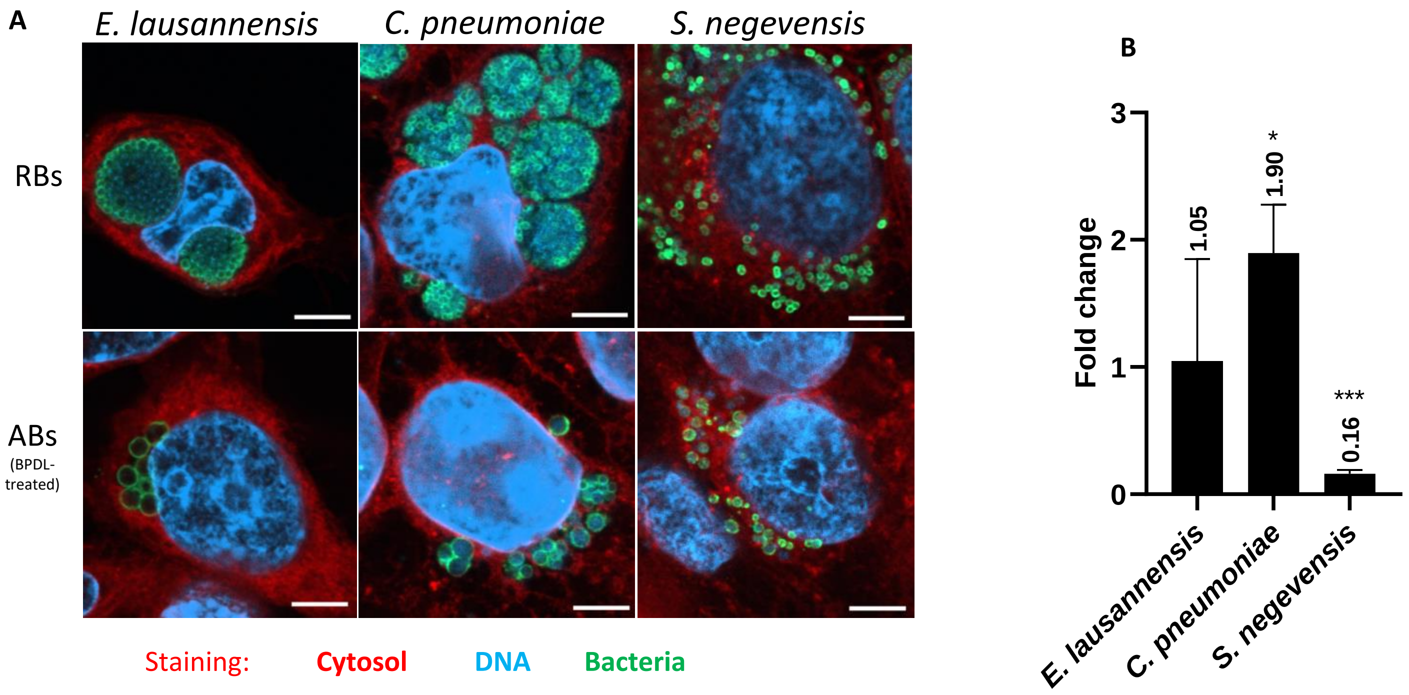

Supplementary Data 9: Aberrant body morphology and *wcw_0502* homologs expression upon iron starvation of *E. lausannensis, S. negevensis* or *C. pneumoniae*–infected cells: A) Immunofluorescence images comparing the morphology of *E. lausannensis, S. negevensis* and *C. pneumoniae* in Hep-2 cells, treated or not with BPDL, 8 hpi. Scale bar: 5 µm **B)** Expression profile of *wcw_0502* homologs in ABs of *E. lausannensis, C. pneumoniae* and *S. negevensis* induced by iron starvation compared to untreated RBs, 24 hpi. Each error bar represents standard deviation of three biological replicates. Asterisks show significance from unpaired t-test, where $p<0.05(*)$, $<0.01(**)$, and $<0.001(***)$.
